# Impact of COVID-19 Pandemic on the Psychological Status of Pregnant Women

**DOI:** 10.7759/cureus.12875

**Published:** 2021-01-23

**Authors:** Prasuna Jelly, Lisa Chadha, Navjeet Kaur, Suresh Sharma, Rakesh Sharma, Shine Stephen, Jitendra Rohilla

**Affiliations:** 1 College of Nursing, All India Institute of Medical Sciences, Rishikesh, Rishikesh, IND; 2 College of Nursing, All India Institute of Medical Sciences, Jodhpur, Jodhpur, IND; 3 College of Nursing, All India Institute of Medical Sciences, Bhubaneswar, Bhubaneswar, IND; 4 Psychiatry, All India Institute of Medical Sciences, Rishikesh, Rishikesh, IND

**Keywords:** coronavirus infection, pandemics, pregnancy, psychology, anxiety

## Abstract

Introduction

Pregnancy is a beautiful phase in every woman's life in which she undergoes several physical and psychological transformations. The level of stress and anxiety may increase due to a sudden outbreak of contagious diseases.

Objective

To evaluate the psychological status of pregnant women during the coronavirus disease-2019 (COVID-19) outbreak.

Materials and methods

A cross-sectional survey was conducted from July 15, 2020, to September 15, 2020, in Dehradun, Haridwar, and Nainital districts of Uttarakhand, India. A total of 333 pregnant women were surveyed through an online platform. The psychological impact of the COVID‐19 pandemic was measured using the Impact of Event-Revised (IES-R) scale, and anxiety levels were measured using the Generalized Anxiety Disorder-7 (GAD-7) scale. Data were analysed using descriptive and inferential statistics.

Results

The survey results revealed that around three-fourths (73.6%) of the pregnant women reported minimal psychological impact, with a mean IES-R score of 16.93±11.23, whereas 69.4% of respondents had a minimal level of anxiety, with a mean GAD-7 score of 3.09±3.73.

Multivariate linear regression found a positive association between psychological impact and gestational age, occupation, religion, locality, conception, history of abortion (p<0.05). Also, the level of anxiety was significantly associated with education, occupation, monthly income, religion, marital and family support, history of mental illness (p<0.01), conception type, and awareness regarding COVID-19 (p<0.05).

Conclusion

Psychological impact and anxiety levels were found to be minimal in pregnant women residing in Uttarakhand. Early identification of high-risk women is important to formulate necessary strategic planning to reduce the complications associated with maternal psychological stress on developing fetus.

## Introduction

Pneumonia of unknown etiology was initially reported in Wuhan on December 31, 2019, located in the Hubei province, in the central part of China [1]. On January 30, 2020, the World Health Organization (WHO) identified Severe Acute Respiratory Syndrome-Coronavirus (SARS-CoV-2) as the most infectious respiratory disease with a high rate of human-to-human transmission called coronavirus disease 2019 (COVID-19) and declared as a global health emergency. As it continued to spread worldwide, affecting around 114 countries and killing more than 4,000 people, WHO officially declared it a pandemic on March 11, 2020 [2].

Pregnancy is one of the most beautiful experiences in a woman's life, in which she undergoes many physical and psychological changes. It gives pride to women to be on the way to becoming new parents, which adds some extra essence to a woman's life. Women may undergo some kind of stress and anxiety during the pregnancy, mainly associated with the fear of potential adverse obstetrical outcomes such as fetal death or fetal abnormalities [3].

The sudden outbreak of COVID-19 with the surge in the number of infected cases and deaths has caused a serious and critical impact on public health, including mental health, which is primarily associated with the symptoms of mental illness such as loneliness, depression, sleep disturbance, anger, etc., not only in health care professional but also among the general public [4]. The risk of depression is higher among pregnant women mainly due to sudden and extreme changes in the daily routine, self-Isolation, social distancing, and stress due to limited follow-up by a physician, and birthing options [5]. The pandemic has also increased the psychological distress, anxiety, and stress among pregnant women due to their uncontrolled worry about their health, the safety of their unborn baby, and quandary about the use of vaccines or medications for prophylaxis and treatment [6]. The intra-uterine development of a fetus entirely depends on the physical as well as the psychological health status of the mother during pregnancy [7].

A recent study indicates that pregnant women are more prone to anxiety and depression during the COVID-19 outbreak, which is mainly associated with their age, cultural status, and the duration of pregnancy [7]. Unrelenting and prominent prenatal anxiety and depression symptoms may increase the risk of postnatal depression, as well as postpartum infection and illness rates [8]. It may affect the maternal mood and development of the fetus due to alteration in physical activity, nutrition, and sleeping pattern in the antenatal period [9]. The risk of miscarriage, preterm labor, lower birth weight, and lower Apgar scores at birth is also associated with prenatal anxiety and depressive symptoms [10]. A mother having a high level of stress during her antenatal period is more likely to induce cognitive and behavioral problems in the baby due to changes in the brain structure [11]. These lifelong psychological and neurological effects focus on the utmost need for effective strategic planning to reduce the impact by targeting maternal stress and providing psychological support that may prevent negative outcomes for both the woman and her baby [12].

The graph of COVID-19 is exponentially increasing daily, but the impact of transmission on the mother's and baby's wellbeing is still not very sure. Uttarakhand is located in India's northern part across the Himalayas, *where the current status of COVID-19 is significantly less (0.42%) compared to the total cases (17,52,171) of India* [13]. It is a primary responsibility to support one of the vulnerable groups of society - pregnant women - to access safe maternity services and early detection and treatment of ailments [14]. Currently, there is insufficient information available on the psychological impact of COVID-19 on the mental health of pregnant women in Uttarakhand. So, the present study aimed to survey pregnant women to evaluate psychological effects and anxiety during the COVID-19 pandemic.

## Materials and methods

Study design

This cross-sectional online survey was carried out using Google Forms application (Google LLC, Mountain View, CA, USA) in the Hindi language and the web link was circulated through the WhatsApp messenger app (Facebook, Inc., Menlo Park, CA, USA) to assess the psychological impact and anxiety level among pregnant women due to sudden outbreak of COVID-19.

Participants

All the pregnant women without any history of psychiatric illness and not diagnosed with COVID-19 were included in the survey. The details of the pregnant mothers were collected from the Primary Health Centers (PHCs) and Community Health Centers (CHCs) of Dehradun, Haridwar, and Nanital districts through Accredited Social Health Activist (ASHA) workers. A total of 21 ASHA workers were contacted from Dehradun (9), Haridwar (8), and Nanital (4) districts. A total of 798 pregnant mothers' contact details were received. Telephonic calls were made to them to explain the objectives of the study and to obtain the preliminary data regarding pregnancy status and access to an android phone. Out of them, 550 women agreed to participate in the study, 44 women's contact numbers were not working, and 204 refused to participate in the study. The web link was sent to the 550 pregnant women, out of which 333 completed the survey. In the present study, the convenience sampling method was adopted. The data collection was carried out from July 15, 2020, to September 15, 2020.

Based on the estimated population size of 2,500 pregnant women in the selected geographical area, 95% confidence level, and 5% margin of error, it was estimated that the required sample size will be 333 participants for this study.

Ethical considerations

Ethical approval was obtained from the institutional ethics committee vide with letter no. AIIMS/IEC/20/ dated August 22, 2020. The participants were well informed about the purpose of the survey. An informed electronic written consent was obtained from each participant before they responded to the online survey questions. Confidentiality of information and anonymity of the participants was maintained. 

Study tools

The online survey was conducted using a standardized tool. The first part of the study questionnaire consisted of socio-demographic characteristics of participants, including age, parity, gestational age, family, monthly income, conception, history of abortion, and knowledge regarding COVID-19. The second part of the survey comprised self-report questionnaires from the standardized tool (Impact of Event-Revised [IES-R] scale and Generalized Anxiety Disorder-7 [GAD-7] scale) to assess the psychological impact and anxiety due to COVID-19. The total time taken to fill all the forms was 15-20 minutes.

The IES-R scale, developed by Andreas Maercker and Matthias Schützwohl in 1998, was used to measure the effect of routine life stress, everyday traumas, and acute stress during the COVID-19 pandemic [15]. The IES-R is a short, easily administered self-report questionnaire containing 22 items. Each item is rated on a 5-point scale ranging between 0 (not at all) and 4 (extremely), reflecting the extent to which a stressful life event or a problem bothered the respondent for the past seven days. The items that compose the scale include eight for intrusion symptoms, eight for avoidance and numbing symptoms, and six for arousal symptoms [16]. The higher the score, the higher is the psychological impact, with a maximum score of 88, which indicates the worst PTSS (post-traumatic stress syndrome) state. According to Creamer et al., the psychological impact of IES-R score was classified as normal (0-23), mild (24-32), moderate (33-36), and severe (>37) [17,18]. Also, this tool is free to use and has been utilised by various studies [19,20] in the Indian population to assess psychological impact.

Generalized Anxiety Disorder (GAD-7) is a self-reported, seven-item anxiety scale developed by Spitzer et al. to measure the frequency of general anxiety symptoms that occurs within the past two weeks [21]. It is an online, free-to-use tool, also available in the Hindi language, and has been applied in various research settings and populations. It is a 4-point Likert scale ranging from 0 (never) to 3 (nearly every day). The total score ranges from 0-21: without anxiety symptoms (0-4), with mild anxiety symptoms (5-9), with moderate anxiety symptoms (10-14), and with severe anxiety symptoms (15-21). The increasing scores indicating more severe functional impairments as a result of anxiety [21].

Statistical analysis

The collected data was organized by Google Sheets (Google LLC, Mountain View, CA, USA) and transferred to Excel sheets (Microsoft Corporation, Redmond, WA, USA) and all statistical analyses were performed by using Statistical Package for Social Sciences (SPSS) version-20 (IBM Corp., Armonk, NY, USA). Descriptive statistics were calculated for socio-demographic characteristics and were presented using frequency and percentage. Anxiety scores and psychological impact scores were also expressed in frequency and percentage. The Chi-square test was used to determine the correlation between IES-R and GAD-7 scores.

Multivariate regression analysis was employed to assess the association of psychological impact and anxiety with the socio-demographic characteristics of pregnant women. A p-value of <0.05 was considered statistically significant.

## Results

Characteristics of survey respondents

The response rate was 60.5% (550/333) in the current study. The majority (78.7%) of the participants were in the age group of 21 to 30 years. More than half were primigravida (61.9%) and in the third trimester of pregnancy (53.5%). Most of the subjects (88.9%) claimed to get family support during their pregnancy (Table 1).

**Table 1:** Socio-demographic profile of pregnant women (N=333)

Variables	Frequency	%
Age		
Less than 20 years	0	0
21-30 years	262	78.7
More than 30 years	71	21.3
Education		
Illiterate	20	6.0
Primary school	69	20.7
Secondary school	143	42.9
Graduate and above	101	30.3
Occupation		
Homemaker	191	57.4
Business	26	7.8
Private job	33	9.9
Government job	48	14.4
Health care worker	35	14.4
Monthly income		
Rs. Less than 5,000	75	22.5
Rs.5,001-10,000	111	33.3
Rs. 10,001-15,000	42	12.6
Rs.15,001-20,000	61	18.3
Rs. 20,001 and above	44	13.2
Religion		
Hindu	291	87.4
Muslim	21	6.3
Christian	9	2.7
Others	12	3.6
Residence		
Urban	159	47.7
Rural	101	30.3
Semi-urban	73	21.9
Family type		
Nuclear	142	42.6
Joint	191	57.4
Family support		
Yes	296	88.9
No	37	11.1

A large number (77.8%) of participants did not have any co-morbidities in pregnancy. A vast number (97%) of participants reported not having any history of mental illness and had a spontaneous conception (96.4%). Almost three-fourth (74.2%) did not have any previous history of abortion. All the study participants were aware of COVID-19 (Table 2).

**Table 2:** General health and pregnancy profile of pregnant women (N=333) HTN: hypertension; DM: diabetes mellitus; COVID-19: coronavirus disease 2019

Pregnancy profile	Frequency	%
Parity		
Primigravida	206	61.9
Multigravida	127	38.1
Gestational age		
1^st^ trimester	43	12.9
2^nd^ trimester	112	33.6
3^rd^ trimester	178	53.5
Conception type		
Spontaneous	321	96.4
Infertility treatment	12	3.6
History of abortion		
Yes	86	25.8
No	247	74.2
General health profile	frequency	%
Presence of co-morbidities		
Yes	74	22.2
No	259	77.8
If yes specify		
Gestational HTN	17	5.1
Gestational DM	11	3.3
Thyroid	29	8.7
Other	17	5.1
History of any mental illness		
Yes	10	3
No	323	97
Awareness regarding COVID-19		
Yes	333	100
No	0	0
If yes, specify source		
Family/friends	92	27.6
Newspaper/magazine	35	10.5
Social media	103	30.9
News/radio	39	11.7
Health care worker	64	19.2

Psychological impact

The psychological impact of the COVID-19 outbreak was measured by the IES-R scale, which demonstrated that the mean IES-R score was 16.93 ± 11.23. The majority of respondents (73.6%) had a minimal psychological impact (IES-R score of 0-23) from the COVID-19 outbreak. About one-fifth of the pregnant women (18.3%) had a mild psychological impact (IES-R score of 24-32), whereas 3% and 5.1% of them reported having a moderate and severe psychological impact with IES-R score of 33-36 and >36, respectively (Table 3).

**Table 3:** Psychological impact and level of anxiety in response to COVID-19 outbreak (N=333) COVID-19: coronavirus disease 2019

Psychological impact (Scores)	Frequency (%)	Anxiety (Scores)	Frequency (%)	r (p-value)
Normal (0-23)	245 (73.6)	No anxiety (0-4)	231 (69.4)	0.5 (<0.01)
Mild (24-32)	61 (18.3)	Mild (5-9)	80 (24.0)	
Moderate (33–36)	10 (3.0)	Moderate (10-14)	18 (5.4)	
Severe (≥37)	17 (5.1)	Severe (15-21)	4 (1.2)	
Mean ±SD	16.9±11.2	Mean ±SD	3.09±3.7	

Anxiety level

The anxiety level due to the COVID-19 outbreak was measured by the GAD-7 scale, which displayed the mean GAD-7 score as 3.09 ± 3.73. The majority of respondents (69.4%) had minimal anxiety levels (GAD-7 score of 0-4) due to the COVID-19 outbreak, while 24% and 5.4% of the participants experienced mild and moderate anxiety levels with GAD-7 score of 5-9 and 10-14, respectively. However, 1.2% of the respondents rated the anxiety level as severe (GAD-7 score of 15-21) (Table 3).

Association of psychological impact with anxiety score

A significant association was traced between GAD-7 and IES-R (r=0.500, p<0.05) (Table 3).

Association of psychological impact scores with socio-demographic variables 

Multivariate linear regression suggested a significant association between gestational age (p<0.01), occupation (p<0.01), religion (p<0.01), locality (p=0.05), conception (p<0.01), history of abortion (p=0.01), and awareness regarding COVID-19 (p=0.03) with psychological impact scores (Table 4).

**Table 4:** Association of psychological impact score with socio-demographic characteristics of pregnant women (N=333) COVID-19: coronavirus disease 2019

Variables	Unstandardized Beta	Standardized Beta	95% CI	p-value
Age	0.087	0.046	0.076-1.073	0.435
Parity	0.050	0.032	-0.597-0.142	0.541
Gestational age	0.287	0.260	0.093-0.651	0.000
Education	0.024	0.026	-0.169-0267	0.626
Occupation	0.104	0.197	0.032-0.284	0.000
Monthly income	-0.031	-0.054	-0.059-0.216	0.314
Residence	-0.101	-0.103	-0.108-0.341	0.045
Family	0.110	0.070	-0.14-0.594	0.181
Marital and family support	0.033	0.013	-0.451-0.78	0.809
Presence of comorbidities	-0.144	-0.077	-0.796-1.355	0.548
Comorbidities	0.063	0.097	-0.093-0.631	0.436
History of mental illness	0.306	0.067	-0.755-1.369	0.197
Conception	0.793	0.190	0.505-2.59	0.001
History of abortion	0.248	0.139	-0.11-0.774	0.012
Awareness regarding COVID-19	-0.068	-0.126	-0.272-0.004	0.028

Association of anxiety scores with socio-demographic variables

In the multivariate linear regression, there was a significant association between parity (p=0.040), education (p=0.002), occupation (p<0.01), monthly income (p<0.01), religion (p<0.01), marital and family support (p<0.05), history of mental illness (p<0.01), conception (p<0.01), awareness regarding COVID-19 with anxiety scores (Table 5).

**Table 5:** Association of anxiety score with socio-demographic characteristics of pregnant women (N=333) COVID-19: coronavirus disease 2019

Variables	Unstandardized Beta	Standardized Beta	95% CI	p-value
Age	-0.087	-0.055	-0.173-0.181	0.322
Parity	0.134	0.101	-0.047-0.216	0.040
Gestational age	0.095	0.104	0.008-0.206	0.054
Education	0.117	0.157	-0.015-0.14	0.002
Occupation	0.093	0.212	0.042-0.131	0.000
Monthly income	-0.090	-0.188	-0.115- -0.017	0.000
Residence	0.005	0.006	0.031-0.241	0.905
Family	0.063	0.048	-0.065-0.195	0.328
Marital and family support	-0.244	-0.119	-0.455- -0.017	0.025
Presence of comorbidities	-0.053	-0.034	-0.4770.288	0.780
Comorbidities	0.046	0.086	-0.109-0.149	0.471
History of mental illness	-1.412	-0.373	-1.576- -0.821	0.000
Conception	0.688	0.198	0.098-0.84	0.000
History of abortion	0.043	0.029	-0.07-0.244	0.582
Awareness regarding COVID	-0.085	-0.191	-0.126- -0.027	0.001

## Discussion

This was a cross-sectional survey to assess the psychological impact and anxiety in pregnant women during the COVID-19 pandemic in Uttarakhand. The results of the present study showed that the COVID-19 outbreak had a minimal-to-mild psychological impact on pregnant women with a mean IES-R score of 16.93±11.23. Around 73.6% of pregnant women have reported minimal psychological impact, whereas 18.3% had a mild psychological impact, 3% had moderate and 5.1% reported severe psychological impact due to the COVID-19 outbreak.

The findings of the study suggest that the COVID-19 pandemic is not causing a serious effect on the psychological status of the pregnant women residing in Uttarakhand, but it is important to perform effective strategic planning to identify the pregnant mothers who are at risk of developing psychological distress and take appropriate actions to prevent aftereffects. The study results are incongruent with similar studies from other nations.

A similar study in Italy found that the COVID-19 outbreak caused a moderate psychological impact on pregnant women, and around 53% of respondents reported severe psychological impact [22]. A study done in Bosnia and Herzegovina and Serbia reported that around that 34.2% of respondents experienced a severe psychological impact, 9.9% experienced moderate, and 23.0% of respondents had mild psychological impact due to the COVID-19 outbreak [23]. Another study done in China showed that the mean IES score of pregnant women was higher than the present study, with 67.1% of pregnant women had an IES ⩾26, indicating moderate-to-severe psychological impact during the outbreak of the COVID-19 pandemic [24]. Most of these studies were conducted during the initial phase of the COVID-19 outbreak in the country and it could be the reason for escalated psychological impact in pregnant mothers, which might have changed over time. 

A large number (69.4%) of pregnant mothers reported minimal level of anxiety due to sudden outbreak of COVID-19, whereas 24% reported mild anxiety level, 5.4% reported moderate anxiety level, and 1.2% reported severe level of anxiety. The prevalence of anxiety during a pandemic would be due to the fear of not receiving necessary prenatal care [25].

The anxiety levels are mainly associated with stress, psychological distress caused by the outbreak of COVID-19, which has led to serious psychological challenges for pregnant women causing both long-term and short-term effects on the baby.

Similar to the present study findings, a study from Belgium reported that 47% of respondents had a minimal level of anxiety, 39.4% mild, 8.4% moderate, and 5.2% severe level of anxiety experienced by pregnant mothers [26]. Whereas a study from Canada reported that a higher proportion (57%) of pregnant women reported a higher level of anxiety symptoms, mainly associated with the fear of transmission to a baby as well as concern about poor prenatal care, relationship problem, and social isolation due to the COVID-19 pandemic [25]. The pandemic had caused a significant increase in the level of anxiety among pregnant women, which is mainly associated with the over concern for their older relatives, children, followed by their unborn baby [27]. In another study from Italy, 46% of pregnant women reported a high level of anxiety due to fear of vertical transmission of the disease to a baby as assessed by a Visual Analogue Scale (VAS) anxiety score ≥of 50 [22]. The study results in Colorado reported 12% of respondents had high depressive symptomology and around 60% reported moderate or severe anxiety symptoms [28].

The present study results suggested that gestational age, occupation, religion, residence, method of conception, history of any abortion, awareness regarding COVID-19 were a predictor for higher psychological impact among pregnant mothers in response to COVID-19, whereas parity, education level, occupation, monthly income, religion, marital and family support, history of mental illness, method of conception, and awareness regarding COVID-19 were the factors associated with the level of anxiety among pregnant mothers.

A study from China has reported that pregnant women who were less than 35 years, underweight, primipara, middle-income, full-time working women, and residing in a per capita living area of ≥20m2 experience more depressive and anxiety symptoms [12]. Whereas a study from Italy has reported that higher educational status was significantly associated with an increase in the prevalence of state-trait anxiety inventory (STAI-S) values [29], and another study from China stated that the gestational age is causing more psychological impact on pregnant women as the second trimester of pregnancy has IES >26 than pregnant women in the first and third trimesters of pregnancy [24]. Another study done in Pakistan specified a positive association of selected demographic variables with anxiety and psychologically related symptoms [30].

This study is a cross-sectional survey that lacks a longitudinal follow-up of the pregnant mother to study the effects on the outcome of pregnancy. Another limitation is that the study has used convenience sampling, so coverage of a broad sample would be limited to geographical specific.

## Conclusions

This study examined the psychological impact and anxiety of the COVID-19 outbreak on pregnant women in Uttarakhand. The present study concludes that the majority of pregnant women had a minimal psychological impact and anxiety-related symptoms. However, it is essential to assess the stress and plan effective strategies to reduce the psychological impact of the pandemic on pregnant women by providing psychological support, including clarifying their misconceptions about risks of vertical transmission to the growing fetus, risk of congenital malformations, and route of delivery, which may prevent negative outcomes for both the woman and the fetus.
